# The complete mitochondrial genome of *Trichonephila clavipes* (Araneae: Araneidae)

**DOI:** 10.1080/23802359.2021.1974967

**Published:** 2021-09-19

**Authors:** Nobuaki Kono, Hiroyuki Nakamura, Kazuharu Arakawa

**Affiliations:** a Institute for Advanced Biosciences, Keio University, Tsuruoka, Yamagata, Japan; b Spiber Inc., Tsuruoka, Yamagata, Japan

**Keywords:** Golden silk orb-weaver, spider, Araneidae

## Abstract

*Trichonephila clavipes* (Linnaeus, 1767) is known as a golden silk orb-weaver and belongs to the family Araneidae. *T. clavipes* is one of the few spider species whose genome has been reported and model organism for a molecular biology. Here, we present the complete mitochondrial genome sequence (mtDNA) of *T. clavipes*. The sequence was obtained using a long-read Nanopore technology and corrected with an Illumina technology. The circular genome is 14,902 bp in length, and the AT content was 77.21%. The *T. clavipes* mitochondrial genome contains 13 protein-coding genes (PCGs), 22 tRNA genes, and 2 rRNA genes. The majority of PCGs were found on the heavy strain.

## Materials, results, and discussion

**Figure 1.**
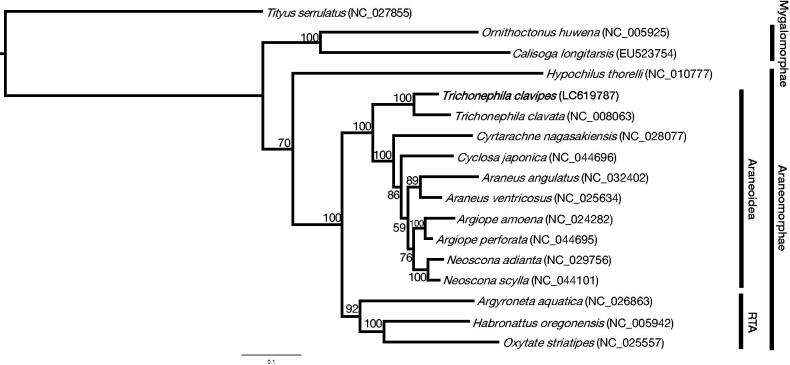
A maximum-likelihood tree was inferred from 17 mitochondrial genomes of arachnids. The phylogenetic tree was constructed based on concatenated amino acid sequences of 13 protein-coding genes. All mitochondrial genome sequences used are obtained from the GenBank database and the accession numbers are shown in parentheses. The RTA is a retrolateral tibial apophysis clade of araneomorph spiders.

Orb-weaving spiders (Orbiculariae) account for 27% of all spiders, the vast majority of which belong to the superfamily Araneoidea (Hormiga and Griswold 2014; World Spider Catalog 2021). *Trichonephila clavipes* and related species formed one of the Araneoidea families, the family Nephilidae, but they were transferred to the family Araneidae, and some Nephila species have been moved to genus *Trichonephila* (Kuntner et al. 2019). Recently, the draft genome of *T. clavipes* has been published and has become the model organism in the molecular biology study of spiders (Babb et al. 2017; Kono et al. 2021) but the mitochondrial genome sequence has yet to be published. The mitochondrial genome sequence of *T. clavipes*, which has undergone various changes in genus and family, provides important information for a phylogenetic relationship analysis of the Orbiculariae at the species level. All the spiders used in this study were adult females, and the *T. clavipes* samples were purchased from Spider Pharm Inc. (Yarnell, AZ, USA). An individual specimen of *T. clavipes* was collected in Citrus County, Florida, USA (28°50′57″N, 82°31′33″W). A specimen and extracted DNA samples were deposited at Institute for Advanced Biosciences, Keio University, Japan (http://www.iab.keio.ac.jp/en/index.html; contact person: Kazuharu Arakawa; e-mail: gaou@sfc.keio.ac.jp) under the voucher number IDV#5884. According to the previous studies (Kono et al. 2019; Kono and Arakawa 2019; Kono et al. 2020) and manufacturer’s protocols, the genomic DNA (gDNA) was extracted from legs and prepared sequence libraries. Long-read sequencing was performed for genome assembly, and Illumina short-reads were used for sequence correction. For long-read sequencing, a high molecular weight (HMW) gDNA was gently extracted from the spider legs using Genomic-tip 20/G (QIAGEN) and purified by over 10 kb size selection with a BluePippin (Sage Science) with a 0.75% Agarose Gel Cassette. The nanopore library was completed following the 1 D library protocol (SQK-LSK108, Oxford Nanopore Technologies). For Illumina short-read sequencing, using the extracted gDNA, the library was prepared with NEBNext Ultra DNA Library Prep Kit for Illumina. Nanopore and Illumina sequencing were performed with the GridION (Oxford Nanopore Technologies) and NextSeq 500 (Illumina, Inc.) instruments and sequenced reads were submitted to DNA Data Bank of Japan (DDBJ), a member of International Nucleotide Sequence Database Collaboration (INSDC), with DRR235161 and DRR235162 in PRJDB10126. The full-length read of the mitochondrial genome was found from the nanopore long-reads with a BLAST search and corrected with the Illumina short-reads. Mitochondrial annotation was performed using the MITOS Web Server (Bernt et al. 2013) and ARWEN v. 1.2 program (Laslett and Canback 2008). Genes were also manually curated based on the closely related species mitochondrial genome sequences. The complete mitochondrial genome of *T. clavipes* was submitted to DDBJ with an accession number LC619787. The complete mitochondrial genome was 14,902 bp in length with AT/GC contents of 77.2 and 22.8%, respectively. Genome annotation represented the 13 protein-coding genes (*cox1* to *cox3*, *nd1* to *nd6*, *nd4l*, *atp6*, *atp8*, and *cob*), 22 transfer RNA genes (tRNAs), and 2 rRNA. The gene composition and orientation of *T. clavipes* were consistent with the mitochondrial genome of *Trichonephila clavata* (NC_008063). Four PCGs (*nd1*, *nd4*, *nd4l*, and *nd5*) were encoded in the light strand, and the remaining nine genes were encoded in the heavy strand. Both rRNAs were encoded on the light strand and located between tRNA^Leu^ and tRNA^Gln^ and separated by tRNA^Val^ gene. The longest overlap was 36 bp in length and located between nd5 and tRNA^Phe^. The 16S rRNA was 1066 bp in length with G + C content of 19.3%, and the 12S rRNA was 693 bp in length with G + C content of 21.4%. The 13 protein-coding genes were used to study the phylogenetic relationship of spiders. These amino acid sequences were aligned with MAFFT version 7.309 (Katoh and Standley 2013), followed by trimAl version 1.2 (Capella-Gutierrez et al. 2009) and RAxML version 8.2.11 (Stamatakis 2014) with 1,000 bootstraps. *Tityus serrulatus* (scorpion) was used as an outgroup. The produced phylogenetic tree was drawn by FigTree version 1.4.3 (http://tree.bio.ed.ac.uk/software/figtree/). The phylogenetic tree represents the genus *Trichonephila* in a different clade from *Araneus* or *Neoscona*, indicating the relationship that once formed the family Nephilidae (Figure 1).

## Data Availability

The data that support the findings of this study are openly available in DDBJ at https://www.ddbj.nig.ac.jp, accession number of LC619787. The associated BioProject, SRA, and Bio-Sample numbers are PRJDB10126, DRX225351, and SAMD00233890, respectively.

## References

[CIT0001] Babb PL, Lahens NF, Correa-Garhwal SM, Nicholson DN, Kim EJ, Hogenesch JB, Kuntner M, Higgins L, Hayashi CY, Agnarsson I, et al. 2017. The Nephila clavipes genome highlights the diversity of spider silk genes and their complex expression. Nat Genet. 49(6):895–903. 28459453 10.1038/ng.3852

[CIT0002] Bernt M, Donath A, Jühling F, Externbrink F, Florentz C, Fritzsch G, Pütz J, Middendorf M, Stadler PF. 2013. MITOS: improved de novo metazoan mitochondrial genome annotation. Mol Phylogenet Evol. 69(2):313–319. 22982435 10.1016/j.ympev.2012.08.023

[CIT0003] Capella-Gutierrez S, Silla-Martinez JM, Gabaldon T. 2009. trimAl: a tool for automated alignment trimming in large-scale phylogenetic analyses. Bioinformatics. 25(15):1972–1973. 19505945 10.1093/bioinformatics/btp348 PMC2712344

[CIT0004] Hormiga G, Griswold CE. 2014. Systematics, phylogeny, and evolution of orb-weaving spiders. Annu Rev Entomol. 59:487–512. 24160416 10.1146/annurev-ento-011613-162046

[CIT0005] Katoh K, Standley DM. 2013. MAFFT multiple sequence alignment software version 7: improvements in performance and usability. Mol Biol Evol. 30(4):772–780. 23329690 10.1093/molbev/mst010 PMC3603318

[CIT0006] Kono N, Arakawa K. 2019. Nanopore sequencing: review of potential applications in functional genomics. Dev Growth Differ. 61(5):316–326. 31037722 10.1111/dgd.12608

[CIT0007] Kono N, Nakamura H, Mori M, Tomita M, Arakawa K. 2020. Spidroin profiling of cribellate spiders provides insight into the evolution of spider prey capture strategies. Sci Rep. 10(1):15721. 32973264 10.1038/s41598-020-72888-6 PMC7515903

[CIT0008] Kono N, Nakamura H, Mori M, Yoshida Y, Ohtoshi R, Malay AD, Pedrazzoli Moran DA, Tomita M, Numata K, Arakawa K, et al. 2021. Multicomponent nature underlies the extraordinary mechanical properties of spider dragline silk. Proc Natl Acad Sci USA. 118(31):e2107065118. 10.1073/pnas.2107065118 PMC8346794 34312234

[CIT0009] Kono N, Nakamura H, Ohtoshi R, Moran DAP, Shinohara A, Yoshida Y, Fujiwara M, Mori M, Tomita M, Arakawa K, et al. 2019. Orb-weaving spider *Araneus ventricosus* genome elucidates the spidroin gene catalogue. Sci Rep. 9(1):8380. 31182776 10.1038/s41598-019-44775-2 PMC6557832

[CIT0010] Kuntner M, Hamilton CA, Cheng R-C, Gregorič M, Lupše N, Lokovšek T, Lemmon EM, Lemmon AR, Agnarsson I, Coddington JA, et al. 2019. Golden Orbweavers ignore biological rules: phylogenomic and comparative analyses unravel a complex evolution of sexual size dimorphism. Syst Biol. 68(4):555–572. 30517732 10.1093/sysbio/syy082 PMC6568015

[CIT0011] Laslett D, Canback B. 2008. ARWEN: a program to detect tRNA genes in metazoan mitochondrial nucleotide sequences. Bioinformatics. 24(2):172–175. 18033792 10.1093/bioinformatics/btm573

[CIT0012] Stamatakis A. 2014. RAxML version 8: a tool for phylogenetic analysis and post-analysis of large phylogenies. Bioinformatics. 30(9):1312–1313. 24451623 10.1093/bioinformatics/btu033 PMC3998144

[CIT0013] World Spider Catalog. 2021. World Spider Catalog. Version 22.0: Natural History Museum Bern. https://wsc.nmbe.ch.

